# Finding order in chaos – nanocrystals in amorphous protein gels

**DOI:** 10.1107/S2053230X21010852

**Published:** 2021-11-01

**Authors:** Matthew W. Bowler

**Affiliations:** a European Molecular Biology Laboratory, 71 Ave des Martyrs, Grenoble, CS 90181 F-38042, France

**Keywords:** nanocrystals, amorphous protein gels

## Abstract

Measurements of protein dense phases reveal the presence of highly ordered protein nanostructures. Such phases may be candidates for structural biology measurements on next-generation instruments for molecules that are difficult or impossible to crystallize.

Methods to coax macromolecules into ordered lattices are bread and butter to the readers of *Acta Crystallographica Section F.* This is far from an easy process, and when it succeeds there is an enormous variety in the size, shape and order found within these crystals (Svensson *et al.*, 2019 ▸). Tens of thousands of trials are often needed to find the components of a supersaturated solution that will induce the formation of crystals; can anything be salvaged from all the failed crystallization experiments? Amorphous material is often found during screening, and can blaze a trail to crystallization, but is there order in the chaos? In this issue, Greene and colleagues (Greene *et al.*, 2021 ▸) add to their discovery that dense phases of ovalbumin contain highly ordered regions (Greene *et al.*, 2015 ▸), by showing the same phenomenon in a number of other proteins.

The dense phases that proteins form during the crystallization process of salting-out have been largely ignored with respect to their macrostructure. The rise of the XFELs (Chapman *et al.*, 2011 ▸; Spence, 2017 ▸), and more recently microED (Mu *et al.*, 2021 ▸; Wolff *et al.*, 2020 ▸), has made the collection of data from ordered structures on the nanometre scale a real prospect. This justifies the investigation of nanocrystals in order to gain atomic details of macromolecules that can be hard to crystallize. By detailed characterization using X-ray and neutron scattering techniques, combined with electron microscopy and modelling, the authors beautifully demonstrate a variety of microstructures that can be formed within protein gels. The researchers investigated three proteins, all adopting different microstructures: RNAse A that forms nanocrystalline sheets; immunoglobulin G, that forms hexagonally packed tubes; and lysozyme, that forms short, ordered chains. Each of these examples show similarities to the packing observed in fully fledged crystals, demonstrating that this phase is likely a step towards large crystals and, more importantly, could be common to this type of ‘amorphous’ protein gel.

While the number of examples is still perhaps limited, this study clearly shows that protein gels tend to contain microstructures. This can therefore be exploited to gain structural insights into systems that have proved difficult to crystallize and may shed light on the processes of crystallization itself. We look forward to seeing the first diffraction images from these fascinating protein phases.

## Figures and Tables

**Figure 1:**
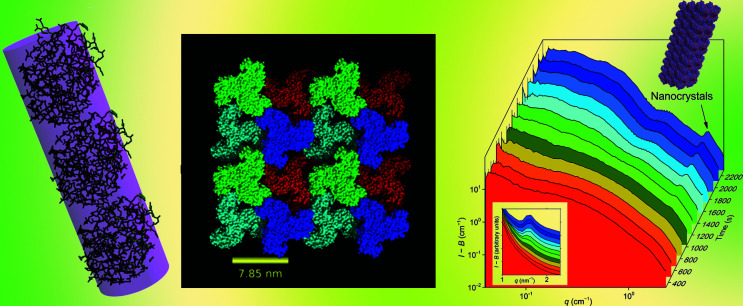
Left to right: lysozyme, RNAse A and ovalbumin. Images reproduced from Greene *et al.* (2021 ▸).

## References

[bb1] Chapman, H. N., Fromme, P., Barty, A., White, T. A., Kirian, R. A., Aquila, A., Hunter, M. S., Schulz, J., DePonte, D. P., Weierstall, U., Doak, R. B., Maia, F. R. N. C., Martin, A. V., Schlichting, I., Lomb, L., Coppola, N., Shoeman, R. L., Epp, S. W., Hartmann, R., Rolles, D., Rudenko, A., Foucar, L., Kimmel, N., Weidenspointner, G., Holl, P., Liang, M., Barthelmess, M., Caleman, C., Boutet, S., Bogan, M. J., Krzywinski, J., Bostedt, C., Bajt, S., Gumprecht, L., Rudek, B., Erk, B., Schmidt, C., Hömke, A., Reich, C., Pietschner, D., Strüder, L., Hauser, G., Gorke, H., Ullrich, J., Herrmann, S., Schaller, G., Schopper, F., Soltau, H., Kühnel, K.-U., Messerschmidt, M., Bozek, J. D., Hau-Riege, S. P., Frank, M., Hampton, C. Y., Sierra, R. G., Starodub, D., Williams, G. J., Hajdu, J., Timneanu, N., Seibert, M. M., Andreasson, J., Rocker, A., Jönsson, O., Svenda, M., Stern, S., Nass, K., Andritschke, R., Schröter, C.-D., Krasniqi, F., Bott, M., Schmidt, K. E., Wang, X., Grotjohann, I., Holton, J. M., Barends, T. R. M., Neutze, R., Marchesini, S., Fromme, R., Schorb, S., Rupp, D., Adolph, M., Gorkhover, T., Andersson, I., Hirsemann, H., Potdevin, G., Graafsma, H., Nilsson, B. & Spence, J. C. H. (2011). *Nature*, **470**, 73–77.

[bb2] Greene, D. G., Modla, S., Wagner, N. J., Sandler, S. I. & Lenhoff, A. M. (2015). *Biophys. J.* **109**, 1716–1723. 10.1016/j.bpj.2015.08.023 PMC4623890 26488663

[bb3] Greene, D. G., Modla, S., Sandler, S. I., Wagner, N. J. & Lenhoff, A. M. (2021). *Acta Cryst.* F**77**, 412–419. 10.1107/S2053230X21009961 PMC8561819 34726180

[bb4] Mu, X., Gillman, C., Nguyen, C. & Gonen, T. (2021). *Annu. Rev. Biochem.* **90**, 431–450. 10.1146/annurev-biochem-081720-020121 PMC9974886 34153215

[bb5] Spence, J. (2017). *IUCrJ* **4**, 322–339. 10.1107/S2052252517005760 PMC5571796 28875020

[bb6] Svensson, O., Gilski, M., Nurizzo, D. & Bowler, M. W. (2019). *IUCrJ* **6**, 822–831. 10.1107/S2052252519008017 PMC6760449 31576216

[bb7] Wolff, A. M., Young, I. D., Sierra, R. G., Brewster, A. S., Martynowycz, M. W., Nango, E., Sugahara, M., Nakane, T., Ito, K., Aquila, A., Bhowmick, A., Biel, J. T., Carbajo, S., Cohen, A. E., Cortez, S., Gonzalez, A., Hino, T., Im, D., Koralek, J. D., Kubo, M., Lazarou, T. S., Nomura, T., Owada, S., Samelson, A. J., Tanaka, T., Tanaka, R., Thompson, E. M., van den Bedem, H., Woldeyes, R. A., Yumoto, F., Zhao, W., Tono, K., Boutet, S., Iwata, S., Gonen, T., Sauter, N. K., Fraser, J. S. & Thompson, M. C. (2020). *IUCrJ* **7**, 306–323. 10.1107/S205225252000072X PMC7055375 32148858

